# Ulnar stress fracture in a softball player

**DOI:** 10.1002/ccr3.2933

**Published:** 2020-05-28

**Authors:** Karen M. Myrick, Scott A. Myrick, Olohiere Ezomo

**Affiliations:** ^1^ School of Interdisciplinary Health and Science Quinnipiac University School of Medicine University of Saint Joseph West Hartford CT USA; ^2^ Quinnipiac University School of Medicine North Haven CT USA; ^3^ Select Sports Medicine South Windsor CT USA

**Keywords:** elite athletes, softball, stress fracture, Ulna

## Abstract

Clinicians need to have a high index of suspicion in overhead athlete with persistent pain. MRI and bone scans are more sensitive than X‐rays in detecting ulnar stress fractures. Increased awareness will improve diagnosis and patient outcomes.

## Graphical abstract

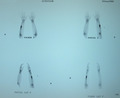

## 1 INTRODUCTION

Ulnar stress fractures are uncommon and can elude immediate diagnosis, therefore prolonging an athlete's discomfort and inability to participate in sports. Mechanisms of injury include increased workload, chronic torsional stress, and repetitive pronation. We present a case of ulnar stress fracture, initially diagnosed as flexor tendinitis in a female high school softball pitcher.

Ulnar stress fractures are very rare.^1^, ^2^, ^3^ Stress fractures are injuries that arise as a result of excessive cyclical force on bones, to which the bone cannot readily adapt to.^3^ Ulnar stress fractures are not common because upper limbs are less likely to have stress fractures because they are not weight bearing. Ulnar stress fractures usually present with medial arm pain that has an extensive array of differential diagnoses with higher prevalence rates. It often eludes diagnosis because initial X‐rays often show no abnormality. This makes the diagnosis of ulnar stress fractures more difficult, leading to misdiagnosis and complications such as severe and overt fractures due to continual weight bearing.

So far, very few articles have been published on ulnar stress fractures. Existing literature shows that patients with ulnar stress fractures are likely to be active athletes that use their upper extremities in excessive balancing, pronation/supination, and rotational motions like softball pitchers^2^, ^4^, ^5^ and baseball players. Ulnar stress fractures have also been described in golfers,^6^ tennis players,^7^ dancers,^8^, ^9^ bowlers,^10^ and baton twirlers.^1^ A rare case was described bilaterally in a patient with rheumatoid arthritis.^3^

In this case report, we discuss an interesting case of right forearm pain in a softball pitcher. Our objectives for this report are to (a) increase awareness about ulnar stress fractures and to (b) highlight a constellation of symptoms that should raise a higher index of suspicion among clinicians for ulnar stress fractures.

## 2 CASE HISTORY AND EXAMINATION

### 2.1 History

A 17‐year‐old, right‐handed softball pitcher with 11 years of softball experience, presented with chief complaints of right knee, elbow, and forearm pain. She was a high‐level athlete, pitching in two leagues for two teams. She was in her senior year of high school and was the recipient of a full scholarship awarded for pitching at a National Collegiate Athletic Association (NCAA) Division 2 college.

At the time of presentation on April 14th, the patient reported that she had noticed her symptoms for approximately 2 months. She described the right elbow pain as a vague discomfort that came on when she was playing in the field and throwing repetitively overhand. She did not feel that this pain was much of a problem, if at all, when pitching windmill style. Of the two complaints, she seemed to feel that the right knee pain was more prominent. There was no history of recent injury to her knee before the onset of symptoms. She had presented with similar knee pain before and was managed for capsular irritation, secondary to plica syndrome. The patient perspective was that the knee was giving her same problems again as the softball season started up. Her symptoms had negatively impacted her ability to play as a pitcher.

She was a nonsmoker, takes no alcohol or recreational drug. Other past surgical history was a torn left knee meniscus repaired with arthroscopic surgery and partial meniscectomy 2 years prior. A typical week for this athlete consisted of the following schedule:

Monday: 2 hours of softball practice, including running, throwing, and drills.
Tuesday: Game day
Wednesday: Practice
Thursday: Game day
Friday: Practice
Saturday: Off
Sunday: Pitching practice with pitching coach

She felt that one of her best sports attributes was her speed while throwing. The only thing that she wanted to improve was her being pain free. On her health survey, she stated that she wanted to avoid tendonitis of the elbow. Unfortunately, she had been diagnosed with tendonitis by a previous caregiver and had commenced a stretching program. She was compliant with the regimen but had no relief. Therefore, her current presentation at our facility.

### 2.2 Clinical examination and findings

On physical examination, the right knee revealed no effusion. There was full range of motion (ROM), some vague discomfort in the superomedial and medial aspects of the patella, a negative flexion pinch test, and a negative Lachman test.

Physical examination of her right upper extremity demonstrated full range of motion and 5/5 strength. Ligamentous stability was noted, and no discomfort was generated with resistance testing. Examination of the right shoulder demonstrated significant capsular tightness with approximately 125 degrees of external rotation in an abducted position, but only 5 to 10 degrees of internal rotation. Internal rotation on the contralateral side was approximately 50 degrees, and an overall arc of 180 degrees of motion was assessed.

X‐ray evaluation of both knees consisted of AP weight bearing, Rosenberg, Sunrise, and lateral views. She also had right elbow X‐rays, AP, oblique, and lateral and posterior views. All X‐rays were normal, with normal bony anatomy, normal joint spacing, and no obvious fractures or dislocations.

The clinical impression at that visit was ongoing discomfort in the right knee, secondary to medial plical syndrome, and posterior capsular tightness in her right shoulder. Treatment options were discussed, and the patient and her father determined that a cortisone injection was their best option. An intraarticular injection in the knee with cortisone was given, and the patient experienced excellent immediate relief of her discomfort. With regard to her elbow and shoulder, she was given a capsular stretching program, and a discussion ensued about her physical examination findings with an athletic trainer and a certified strength and conditioning coach. We recommended that if this did not improve her symptoms over the next couple of weeks, we would send her off for a formal course of therapy.

The patient returned to us on June 1, 6 weeks later, with persistent right forearm (volar) pain despite resolving right elbow and knee pain. It was noted on this visit that the patient was “frustrated” because of the discomfort that she had been experiencing on the volar aspect of her forearm which had now lasted over a year and was recently getting worse. She noted that after a game she had pitched in the previous week, she experienced significant swelling in her forearm. The pain had negatively impacted her ability to play or engage in physical activity.

Physical examination revealed no swelling, ecchymosis, or gross abnormality. She did not experience any discomfort with bony palpation. With muscle testing, she experienced pain with resisted wrist flexion, and pronation, no pain with wrist extension or supination, and a normal hand and elbow examination. A working diagnosis was made of flexor tendonitis. Due to her ongoing discomfort, the recent localization of her symptoms, and the fact that her level of play could not be maintained, we proceeded to obtain an MRI of the forearm to further evaluate the area. We also discussed casting the forearm for a short period of time as an option, and a rest period. She agreed to a MRI but refused immobilization because she had a tournament she wanted to play in the next day.

### 2.3 Differential diagnosis, investigations, and treatment

Differential diagnoses would include flexor tendinitis, stress fracture, and muscle overuse or strain. Flexor tendinitis would be differentiated by pain primarily on the anterior aspect of the elbow that increases with movement of the hand and fingers and typically exacerbated by wrist flexion and pronation on physical examination.^11^

Stress fracture would be differentiated by pain that is unremitting, and by diagnostic imaging.^12^ Stress fractures are increasingly common, but not well documented in the female population.

### 2.4 Investigations

**Figure 1:**
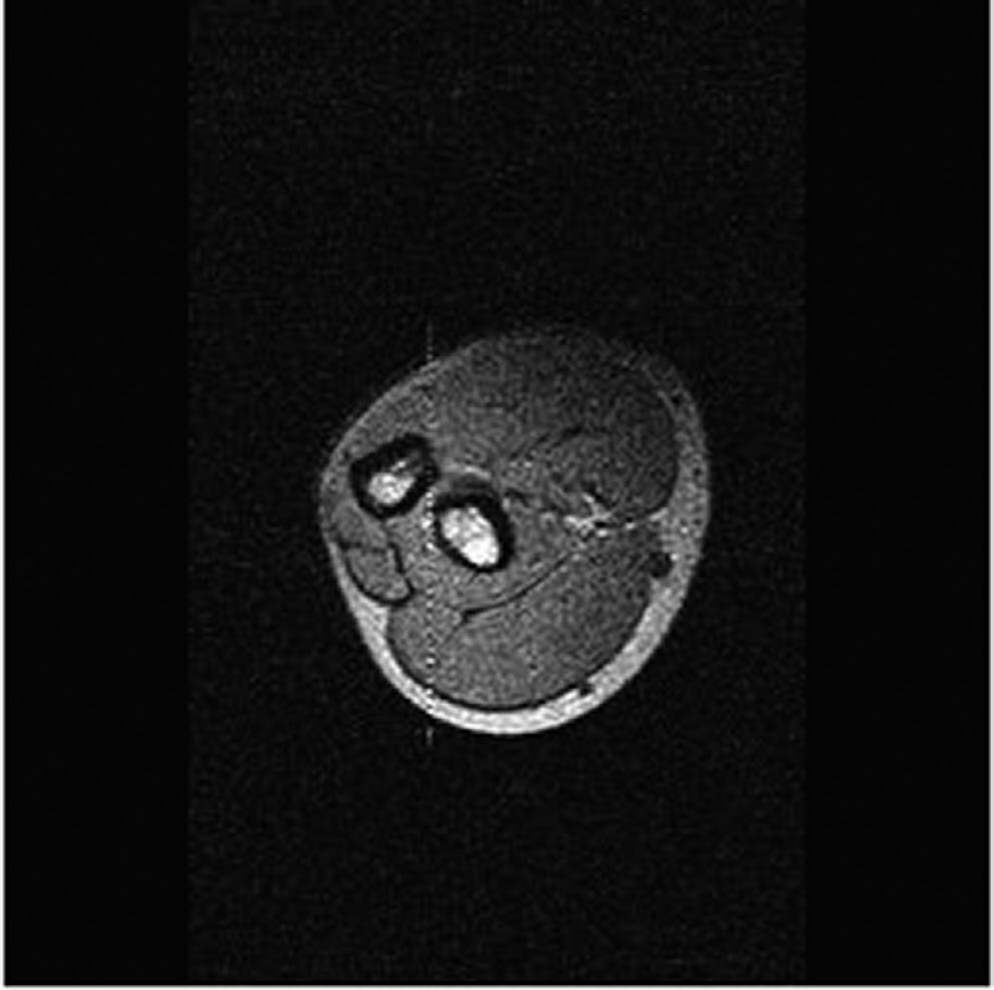
MRI without contrast, T1 image, axial view

**Figure 2:**
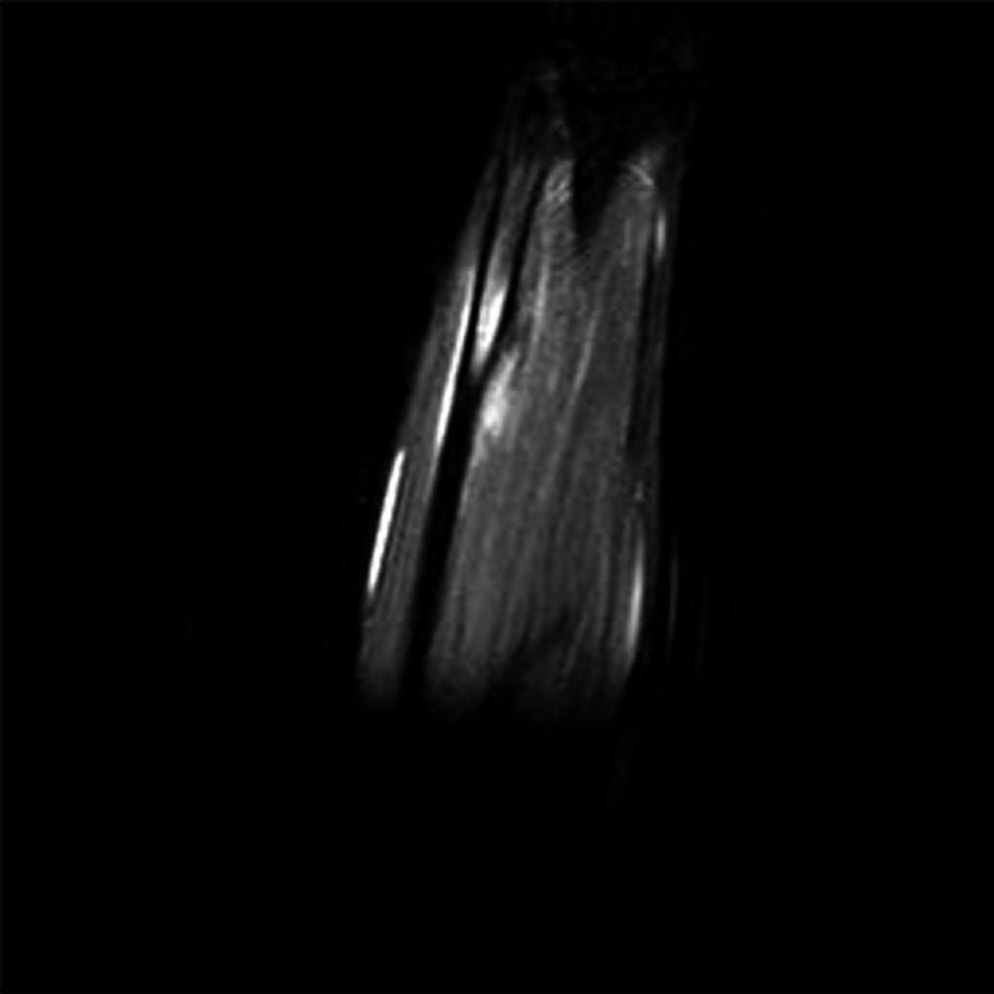
MRI without contrast, T2 image, coronal view

**Figure 3:**
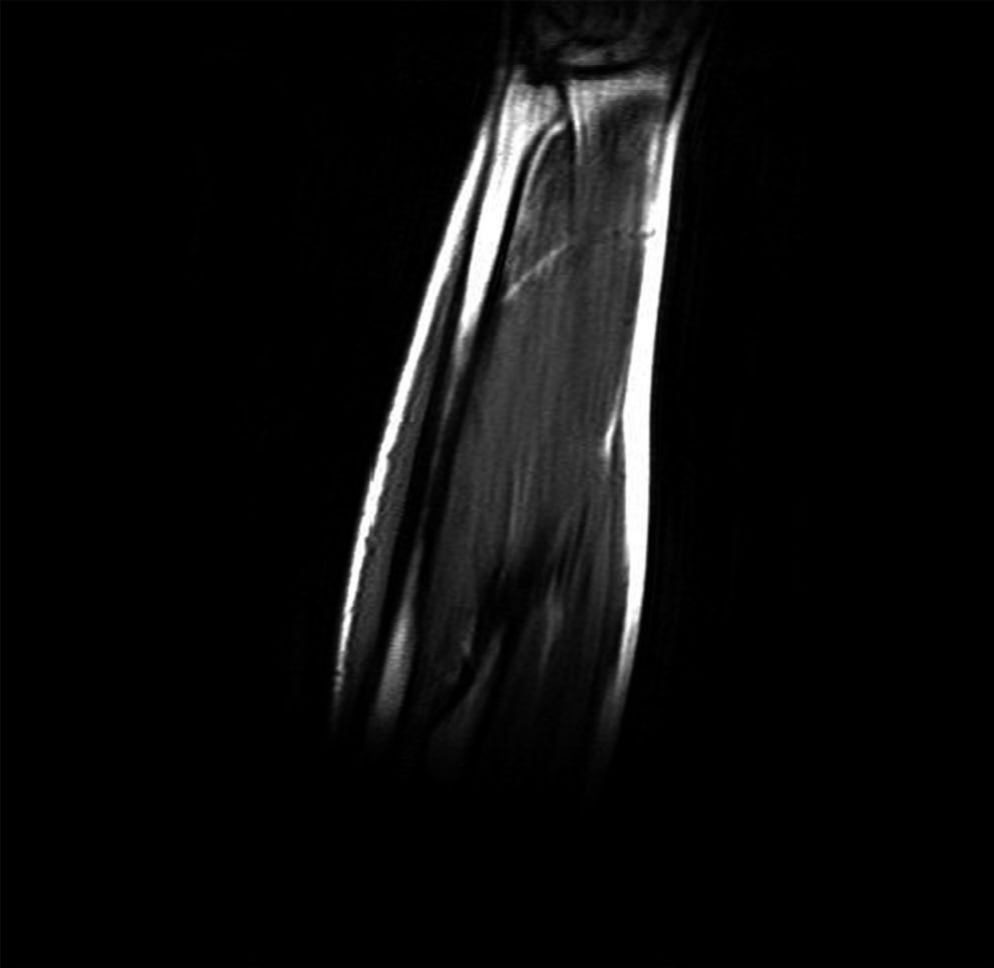
MRI without contrast, T1 image, coronal view

**Figure 4:**
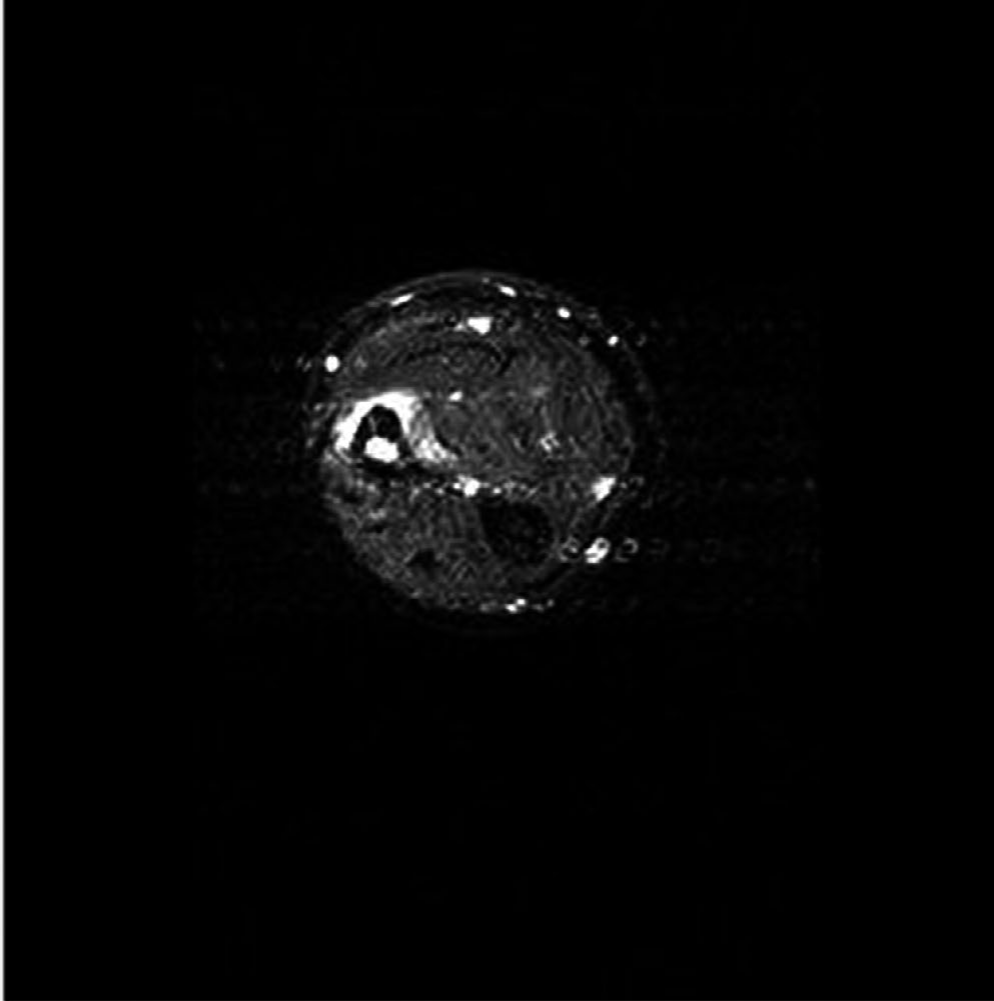
MRI without contrast, T2, Axial view

The MRI without contrast performed on June 8th revealed an abnormal bone marrow signal within the mid to distal third of the ulna, with surrounding periosteal edema and edema in the surrounding musculature immediately adjacent to the affected bone (see Figures 1, 2, 3, 4).

At the follow‐up visit June 15th, a stress fracture was suspected. These results and the present concern were communicated to the patient and family, and a bone scan was ordered.

**Figure 5:**
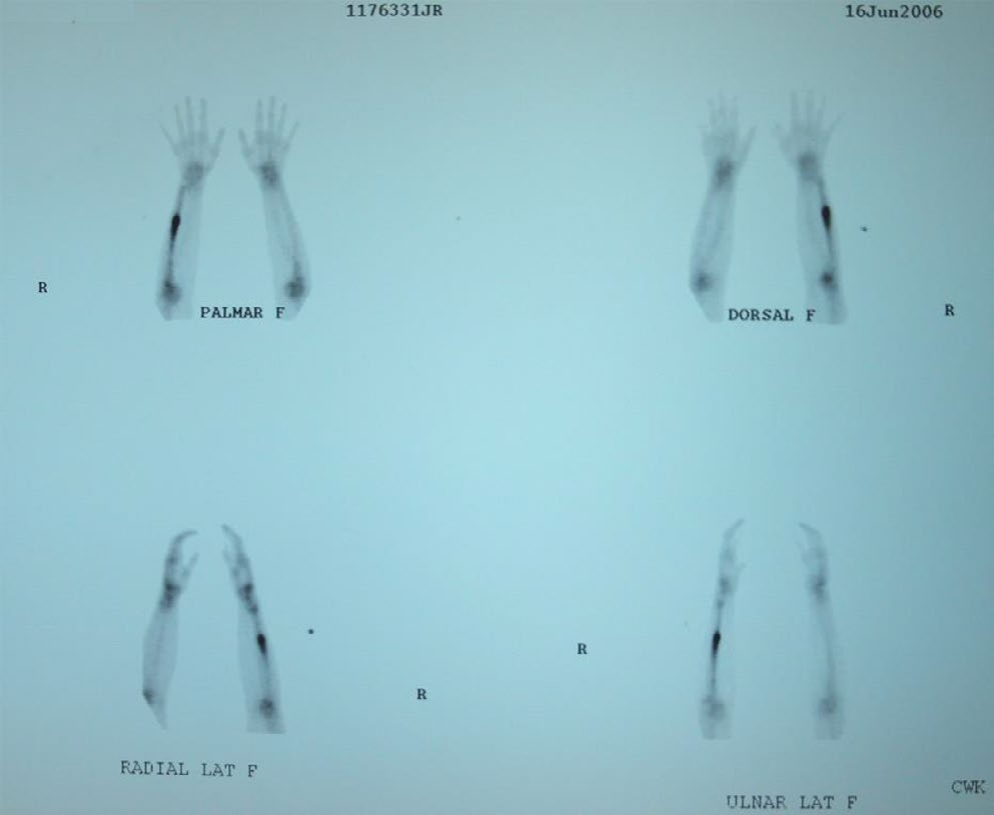
Bone Scan June 16

A bone scan was performed on June16th. The results of the bone scan revealed a specific increase in uptake around the midshaft of the ulna consistent with a stress fracture (see Figure 5).

On June 19th at her follow visit, it was recommended that the patient be casted and immobilized for a period of at least 3 weeks, with assessment and likely continuation of casting for an additional 3 weeks. She was placed into a Muenster cast, and given strict instructions of activity modifications, the outcomes of the injury, and follow‐up.

### 2.5 Outcome and follow‐up

**Figure 6:**
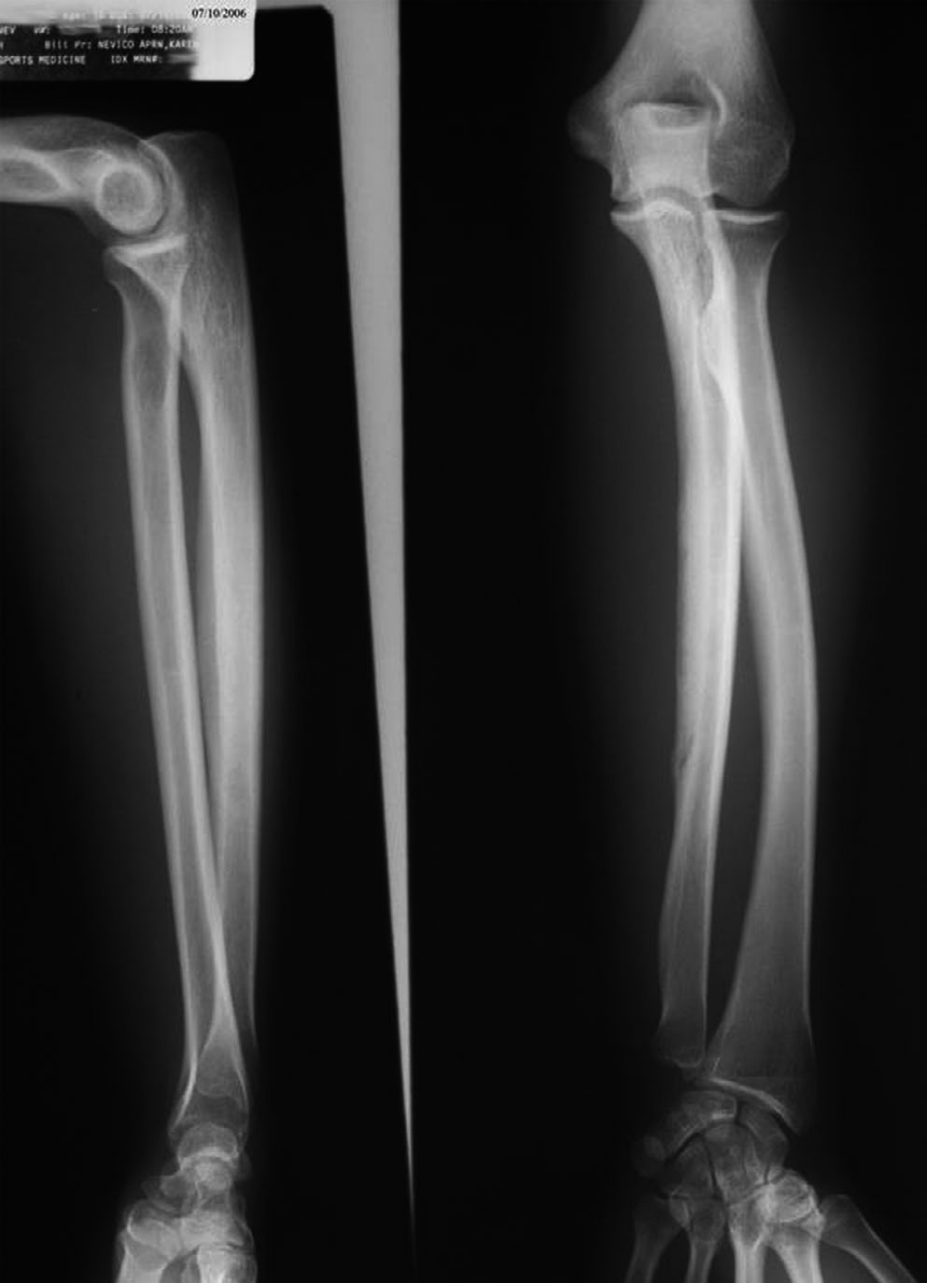
X‐ray July 10

On return visit July 10th, the patient reported that she and her father had taken off her cast three days prior. There was no pain on bony palpation, and X‐rays revealed a remodeling fracture of the mid to distal ulna with callus formation (see Figure 6).

A throwing program was recommended, and she was asked to identify and stop any play or throwing that caused pain. Her next follow‐up visit was scheduled for 2 weeks along with X‐rays.

**Figure 7:**
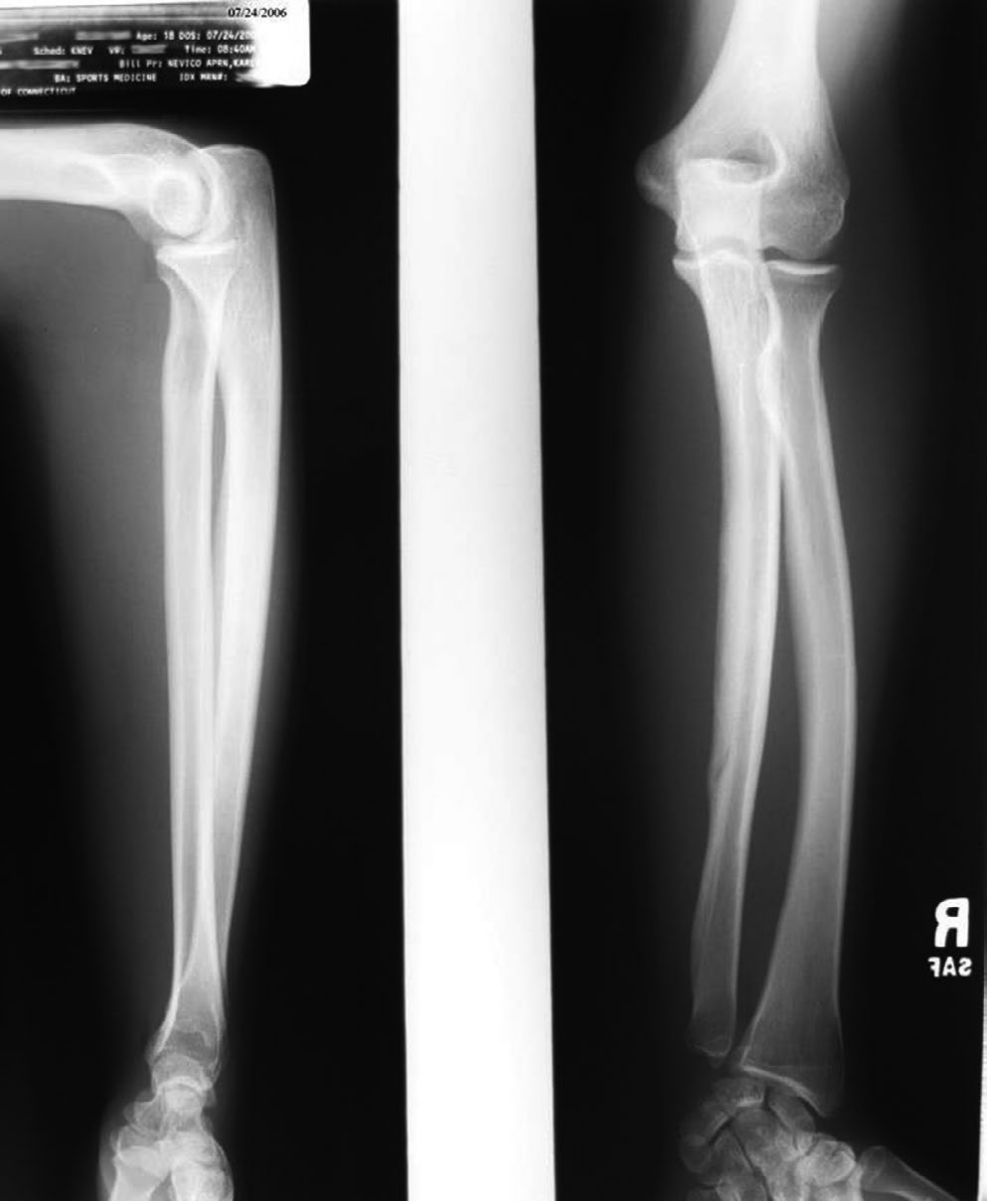
Radiograph 7/24

On follow‐up on July 24th, the patient reported adhering to the throwing program and soreness rules. She was back to her activities at a low level and had just begun the throwing portion of the program and was doing well with it. She admitted to some mild discomfort when she initially removed the cast, but had experienced complete resolution at this point. Her X‐rays revealed continuation of the remodeling of the mid to distal shaft of the ulna (see Figure 7). We reiterated the importance of following the throwing program and soreness rules and asked to see her before she leaves for college.

**Figure 8:**
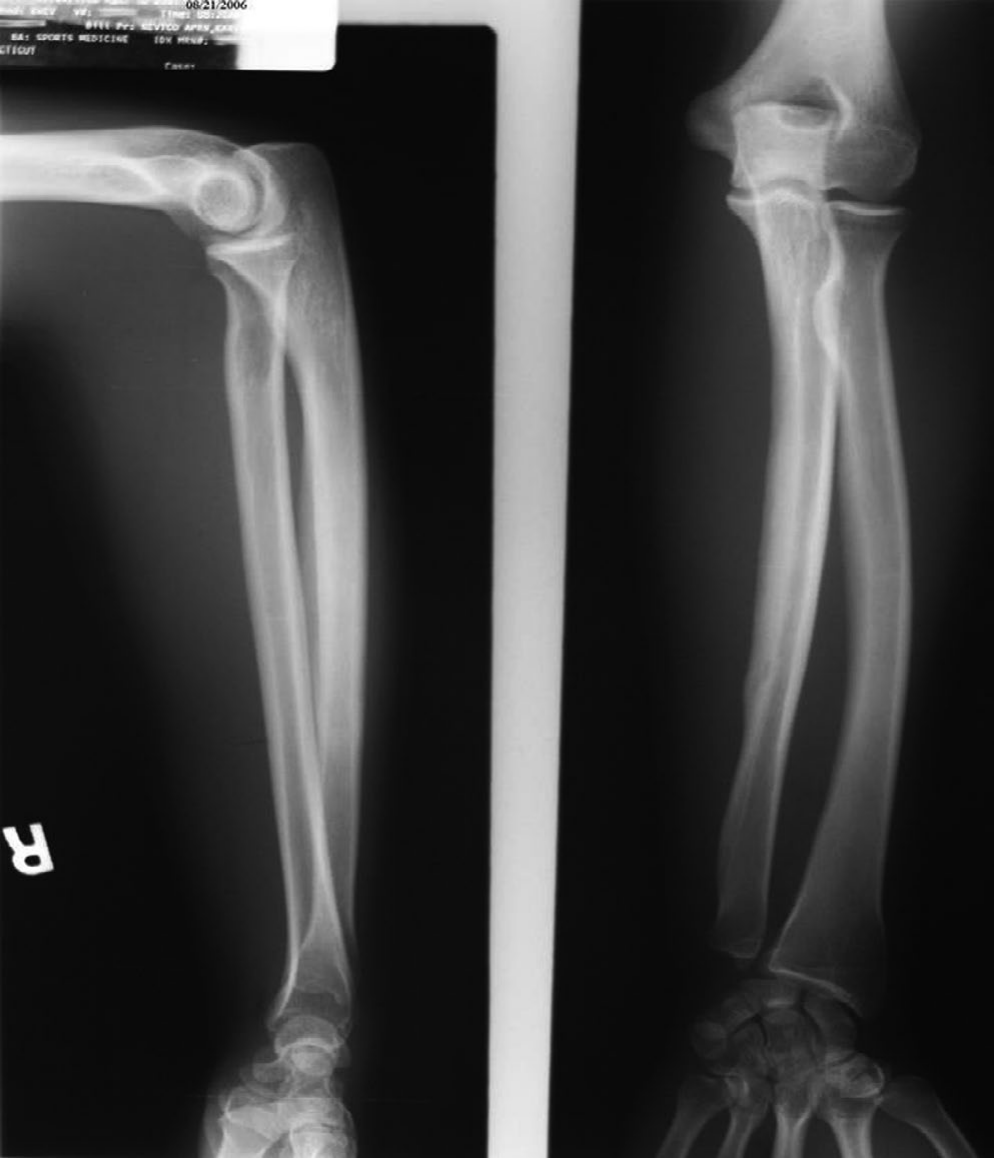
Radiograph 8/21

On August 21st, she was given a full release back to activity, as she was on her way to play for college. She had no pain with any activity and progressed through the throwing program without any issues or problems. Subsequent X‐rays revealed full healing of the stress fracture (see Figure 8).

## 3 DISCUSSION

Our case report shows that ulnar stress fracture is more likely to be diagnosed in patients who are upper extremity athletes. It is more likely to be diagnosed in patients who are upper extremity athletes, associated with severe chronic volar pain that interrupts play/ physical activity and acute swelling worse with physical activity. Symptoms usually do not resolve with conservative management and therapy. It is also better diagnosed with MRI and radioactive uptake as opposed to X‐rays that are less sensitive. Flexor tendonitis of the elbow is a common differential diagnosis. Our patient had pain with resisted wrist flexion and pronation, which is in keeping with flexor tendonitis. However, her pain was on the volar aspect of her forearm and was not exacerbated by movement of the fingers.

Our clinical scenario is similar to other case reports published. Bigosinski et al described a 17‐year‐old softball pitcher with insidious onset of pain in her right forearm. Initial X‐rays done were normal, and a diagnosis of ulnar stress fracture was made weeks after a bone scan and MRI.^4^ Wiltfong et al published a case report of an 18‐year‐old fast‐pitch female softball pitcher that had been previously diagnosed with elbow tendonitis. She subsequently presented after a hard throw with severe forearm pain, swelling, and associated paraesthesias. Imaging studies showed a complete/ overt ulnar stress fracture that had to be repaired surgically.^5^

With regard to location, our patient's stress fracture was located in the mid to distal third of the ulna shaft similar to other published literature.^4^, ^5^ Tanabe et al in their article used CT to observe the cross‐sectional anatomy of the ulna and found that the middle third had the smallest diameter and the thinnest bony cortices thus predisposing this region to the highest risk of stress fractures. During the windmill pitch, the revolving force of the radius exerts a high‐pressure torque on the relatively immobile ulna.^5^, ^13^ Such repetitive forces lead to excessive microtrauma to the ulna and subsequent stress fractures.

Physicians need to have a high index of suspicion for ulnar stress fracture in the following:

- female athletes.
- Patients who are involved in repetitive upper limb actions like athletes.
- Forearm pain > 2 months, usually midshaft.
- Forearm pain on the volar aspect, does not resolve with conservative treatment, is not worse with movement of the fingers.
- Associated acute swelling or increase in pain with activity.

Throwing athletes with upper extremity discomfort that keeps them from play should be evaluated with ulnar stress fracture in mind.^12^ Even though upper extremity stress fractures are rare,^1^, ^14^ and ^15^ and more commonly found in the shoulder girdle than the ulna,^16^, ^17^ the ulna should still be evaluated carefully especially midshaft because of the risk of converting to an overt fracture with continued play.

In our case, we expedited the patient's care by obtaining an MRI, followed by a 3‐phase bone scan. Anderson noted in 2006 that, although a 3‐phase bone scan is more sensitive, an MRI is the study of choice.^18^ Anderson went on to describe that plain films are only 15% sensitive, and over time they are only positive in approximately 50% of patients with stress fractures.^18^ The con of MRI often is that it is more expensive. However, considering the risk of more intraoperative cost, complications, and days lost to long recovery times, the benefit of using an MRI far outweighs the cost.

Interestingly, published literature that reports ulnar stress fractures among softball pitchers are likely to be females like our patient. Even in other sports, the injury is likely to be seen among females. This is extremely important in softball because there are no pitch counts for females compared to their male counterparts. Therefore, they are more likely to pitch more per game and within the season.^19^ Shanley et al in a descriptive study found that average pitch count among injured softball pitchers was 1171 compared to 661 for noninjured pitchers.^20^ It is important that new sport guidelines be established for maximum pitch counts in females per game and per season, especially among high school softballers to prevent joint overuse injuries and stress fractures in them. There is an abundance of research on male baseball pitchers^12^, ^15^, ^21^ and pitch count for male softball pitchers. Currently, there is ruling for a pitch count for youth male pitchers, but not for female softball pitchers. Female softball pitchers are often less considered because they are fewer. As an example, in the 2000 Olympic Games, there were 13 male pitchers on the roster of the 25‐member team, and on the softball team, there were 5 women pitchers on the roster of the 15‐member team.^22^ There is a need to evaluate the demands on female pitchers and to establish a pitch count for these athletes. Future research should be performed also on female windmill pitchers to assess safe upper limits for pitch counts per game and per season.

The report is limited in its generalizability and interpretation because this is a single case report. However, it provides key clinical information that can be further developed to improve clinical diagnosis of ulnar stress fractures.

In conclusion, clinical translational research results applied to patient care greatly improves patient outcomes and prevents worse complications. Through case study review, we can facilitate learning from past experiences, develop problem solving skills, and share management plans. With increased awareness of stress fractures in the upper extremities of female softball pitchers, an improved understanding of the mechanism of injury, and evidence for the best imaging techniques, patient care can be expedited, and early return to play achieved. Ulnar stress fractures are not common but should be highly suspected in throwing/ pitching athletes with persisting volar pain and further workup with MRI and bone scans should be considered early in the patient's management. It is extremely important also that urgent guidelines be established by the World Baseball Softball Confederation and other local softball regulatory bodies, to regulate pitch counts in female softball pitchers to reduce injury and complications.

## CONFLICT OF INTEREST

None declared.

## AUTHOR CONTRIBUTION

All authors contributed to the writing and editing of this manuscript.
